# A Review of Bioactive Compounds against Porcine Enteric Coronaviruses

**DOI:** 10.3390/v14102217

**Published:** 2022-10-08

**Authors:** Cong Duan, Yufeng Luo, Xianming Liang, Xia Wang

**Affiliations:** China Institute of Veterinary Drug Control, Beijing 100081, China

**Keywords:** porcine enteric coronaviruses, diarrhea, host factors, antiviral compounds

## Abstract

Pig diarrhea is a universal problem in the process of pig breeding, which seriously affects the development of the pig industry. Porcine enteric coronaviruses (PECoVs) are common pathogens causing diarrhea in pigs, currently including transmissible gastroenteritis virus (TGEV), porcine epidemic diarrhea virus (PEDV), porcine deltacoronavirus (PDCoV) and swine acute diarrhea syndrome coronavirus (SADS-CoV). With the prosperity of world transportation and trade, the spread of viruses is becoming wider and faster, making it even more necessary to prevent PECoVs. In this paper, the host factors required for the efficient replication of these CoVs and the compounds that exhibit inhibitory effects on them were summarized to promote the development of drugs against PECoVs. This study will be also helpful in discovering general host factors that affect the replication of CoVs and provide references for the prevention and treatment of other CoVs.

## 1. Introduction

Pork is one of the most important meat production and marketing varieties in the world. In the practice of pig production, various intestinal diseases occur frequently, which seriously affects the production performance of pigs and brings great losses to the pig industry. Porcine enteric coronaviruses (PECoVs), including transmissible gastroenteritis virus (TGEV), porcine epidemic diarrhea virus (PEDV), porcine deltacoronavirus (PDCoV) and swine acute diarrhea syndrome coronavirus (SADS-CoV), are common pathogens that cause diarrhea in pigs.

TGEV, PEDV and SADS-CoV all belong to the genus *Alphacoronavirus* of the family *Coronaviridae*. TGEV was first reported in the United States as early as 1946. Although the incidence of TGEV in pig farms is not high, mortality in newborn piglets is almost 100% [1]. PED was first recognized in the UK in 1971, and PEDV was identified as a novel CoV in 1978. The positive detection rate of PEDV in diarrhea samples is generally higher than that of TGEV, and Liu et al. reported that its prevalence rate was 50.21–62.1% [1]. Neonatal piglets infected with PEDV have a mortality of up to 100%. PDCoV is classified in the *Deltacoronavirus* genus. It was first detected in pig rectal swabs during molecular surveillance study and reported in 2012. PDCoV diarrhea was first reported in the United States in 2014. The prevalence rate of PDCoV is lower than that of PEDV but higher than that of TGEV and it generally causes lower neonatal piglet mortality than both. SADS-CoV, also known as swine enteric alphacoronavirus (SeACoV) and porcine enteric alphacoronavirus (PEAV), was first discovered in 2017 in piglets with diarrhea in China. To date, there are not many reports on the detection of SADS-CoV in clinical samples, only in diarrhea samples from the Guangdong, Fujian, Liaoning and Gansu provinces of China [2,3,4,5]. SADS-CoV infection causes up to 90% mortality in piglets aged 5 days or younger, and the mortality falls to 5% in piglets older than 8 days [6]. Currently, there are insufficient commercial drugs for PECoVs. Therefore, it is necessary to summarize the discovered compounds with inhibitory effects on these PECoVs and their inhibitory mechanisms so as to provide a basis for drug development. Given that the efficient replication of viruses depends on the host cell, a summary of the host factors that influence the replication of these PECoVs could help to identify new antiviral targets and even facilitate the discovery of the general replication mechanisms of CoVs.

## 2. Important Host Factors Involved in PECoV Replication

The mechanism of antiviral reagents can be divided into two categories: one targets the viruses themselves and the other targets important host factors involved in the viral life cycle. The replication process of CoVs and the biological functions of multiple CoV proteins have many commonalities, which have been summarized by scholars and will not be elaborated on here. This section mainly reviews the important host factors that affect the life cycle of PECoVs.

### 2.1. Apoptosis

Some viruses escape apoptosis to obtain sufficient time to produce progeny viruses, while some viruses actively induce apoptosis to promote the release and spread of progeny viruses. Studies have shown that apoptosis favors the replication of PEDV, PDCoV and SADS-CoV [7,8,9,10]. PEDV triggers caspase-independent apoptosis through the activation of the mitochondrial apoptosis-inducing factor (AIF) and induces caspase-8- and caspase-3-mediated apoptosis in the late stages of infection [7,8]. The reactive oxygen species (ROS)-p53 pathway participates in PEDV-induced apoptosis [11]. PDCoV causes caspase-dependent intrinsic apoptosis by triggering the release of the mitochondrial cytochrome c into the cytoplasm [9]. SADS-CoV induces both FasL-mediated extrinsic apoptosis and cyclophilin D-dependent intrinsic apoptosis, which are linked by Bid cleavage [10]. The influence of apoptosis on TGEV replication is different from that of the above viruses. The pathway by which TGEV induces apoptosis involves caspase-dependent extrinsic and intrinsic pathways as well as the AIF-mediated caspase-independent pathway, and ROS and p53 regulate these processes [12,13,14]. Nonetheless, blocking the activation of caspases and p53 has no significant effect on TGEV replication.

### 2.2. Autophagy

Autophagy is an evolutionarily conserved pathway in eukaryotic cells and exhibits different effects on different viruses. Guo et al. reported that autophagy negatively regulates TGEV replication [15], while Zhu et al. pointed out that autophagy promotes TGEV infection [16]. Zhu et al. showed that TGEV can induce mitophagy by upregulating the expression of DJ-1, a multifunctional redox-sensitive protein, and the induced mitophagy plays a role in counteracting oxidative stress and apoptosis caused by viral infection. PEDV-induced autophagy is beneficial to viral replication and there is a positive feedback loop between autophagy and the NF-κB signaling pathway during PEDV infection [17]. Zou et al. reported that PEDV ORF3 protein is an autophagy inducer dependent on the endoplasmic reticulum stress (ERS) response [18]. They also indicated that PEDV ORF3 protein stimulates ERS via PERK-eIF2α pathway. However, Wang et al. showed that the knockdown of PERK or the ERS inhibitor 4-phenylbutyrate promotes PEDV replication [19]. PDCoV can trigger autophagy by activating p38, which facilitates viral replication [20]. The interaction between SADS-CoV and autophagy remains to be elucidated.

### 2.3. Mitogen-Activated Protein Kinase (MAPK) Pathway

The MAPK signaling pathway is a ubiquitous signal transduction pathway in mammalian cells and is manipulated by a variety of viruses. Extracellular signal-regulated kinase (ERK), Janus kinase (JNK) and p38 MAPK are three main MAPK pathways that have been extensively studied. They have been proven to function primarily in the post-entry stage of PEDV and PDCoV, such as their involvement in viral biosynthesis and progeny release [21,22,23,24,25]. Gao et al. reported that PEDV can activate p38 through the high mobility group box-1 (HMGB1)/TLR4 pathway [26]. The significance of p38 for TGEV replication is controversial. Dong et al. showed that either p38 inhibitors (BIRB796 and LY222820) or siRNA-mediated knockdown targeting p38 inhibited TGEV propagation, indicating that p38 is necessary for efficient TGEV replication [27]. However, Huang et al. showed that the inhibition of p38 by the inhibitor SB203580 had no significant effects on TGEV gene transcription [13]. This contradiction may be related to differences in p38 inhibitors, and more studies are needed for clarification. In addition, ERK1/2 activation is required for the efficient propagation of SADS-CoV [28].

### 2.4. Cholesterol

Cholesterol is a major component of lipid rafts and affects multiple stages of the viral life cycle, especially the entry process. Both viral and cellular cholesterol are involved in TGEV and PDCoV replication, whereas PEDV requires cellular but not viral cholesterol for efficient replication [29,30,31,32]. The depletion of viral cholesterol does not affect the binding of TGEV to its receptor aminopeptidase N (APN) but rather the post-adsorption step [30]. The attachment and internalization of PEDV requires cellular cholesterol, and both cellular and viral cholesterol are critical for PDCoV attachment and internalization [31,32]. Furthermore, treatment with the cholesterol depletion reagent methyl-β-cyclodextrin (MβCD) prior to infection diminishes SADS-CoV replication [33].

### 2.5. Epidermal Growth Factor Receptor (EGFR)

EGFR belongs to the receptor tyrosine kinases (RTK) family and is widely expressed in many cells. It acts as a promoter for TGEV entry [34,35]. TGEV spike (S) protein can bind to EGFR and activate EGFR under the premise of TGEV binding to APN, leading to cofilin phosphorylation and F-actin polymerization through the PI3K-Rac1/Cdc42-PAK-LIMK pathway early in TGEV infection. Lipid rafts provide a platform for EGFR aggregation and play a crucial role in TGEV attachment and entry. EGFR activation also contributes to PEDV replication, but the stage at which it functions in the PEDV life cycle remains to be revealed. Yang et al. found that PEDV-induced EGFR activation impedes type I IFN responses by activating STAT3, which serves as a negative regulator of type I IFN-mediated antiviral responses [36].

### 2.6. Cellular Receptors for PECoVs

APN is the functional cellular receptor for TGEV, but its role as a receptor for PEDV and PDCoV is controversial [37,38,39,40]. Afterward, Zhang et al. speculated that the transferrin receptor 1 (TfR1) may be a receptor for PEDV [41]. The PEDV S1 protein interacts with the extracellular region of TfR1, and consequent activation of TfR1 endocytosis contributes to PEDV entry. During this process, Src kinase-mediated tyrosine phosphorylation of TfR1 and cellular cholesterol are required for TfR1 internalization. Moreover, heparan sulfate is an attachment factor for PEDV, and sialic acid is an attachment receptor for PDCoV [42,43].

### 2.7. Other Host Factors Involved in PECoV Replication

Transmembrane protein 41B supports the formation of double-membrane vesicles essential for TGEV replication and is involved in TGEV internalization and early-stage replication [44]. Tubulin interacts with TGEV S protein and participates in the incorporation of S protein into virions and the release of virions [45]. Occludin is necessary for PEDV entry as a scaffold in the vicinity of viral entry [46]. Transmembrane protease serine 2 (TMPRSS2) and mosaic serine protease large-form (MSPL) have the characteristics of promoting PEDV S protein cleavage, promoting cell–cell fusion and virus–cell fusion during PEDV infection [47]. In addition, integrin αvβ3, heat shock cognate 70 (Hsc70), heat shock protein 70, JAK2 and STAT3 all play a positive role in PEDV replication [48,49,50,51]. The zinc finger DHHC-type palmitoyltransferase 17 (ZDHHC17 or ZD17) affects SADS-CoV genomic RNA replication, and the DHHC domain of ZD17 with the palmitoyltransferase activity is the key factor [52]. Placenta-associated 8 protein is involved in SADS-CoV subgenomic RNA expression [53]. Ribosomal Protein L18 and Ras Homolog Family Member A, which interact with SADS-CoV M protein, also contribute to viral replication [54].

## 3. Bioactive Compounds with Anti-PECoV Activity

Among the currently reported compounds with antiviral activity against PECoVs, some show inhibitory effects on two or three of TGEV, PEDV and PDCoV, such as ergosterol peroxide extracted from the traditional Chinese medicine *Cryptoporus volvatus*, 25-hydroxycholesterol (25HC) and melatonin. Table 1 summarizes the compounds with anti-PECoV activity and their mechanisms of action. Reports on bioactive compounds that inhibit SADS-CoV are relatively limited. It is necessary to dig into the key factors affecting SADS-CoV replication. Agents with high inhibitory effects on other RNA viruses (especially CoVs with relatively close evolutionary relationships) are valuable references, and more attention should be paid to agents that are effective against multiple CoVs.

### 3.1. Bioactive Compounds against TGEV

Curcumin, the main polyphenolic compound of turmeric, has an inhibitory effect on the early stage of TGEV infection, especially on viral adsorption, and also possesses a direct virucidal effect on TGEV [55]. Hypericin, a biologically active dianthrone compound extracted from St John’s Wort (*Hypericum perforatum* L.), can bind to TGEV 3CLpro and inhibit TGEV replication by suppressing 3CLpro activity [56]. More importantly, the binding domains of hypericin to 3CLpro are highly conserved in α-CoV, implying that hypericin may be a pan-anti-α-CoV compound. (+)-Catechin, a catechin in green tea, has anti-TGEV properties, which are related to its antioxidation [57]. *Polygonum Cillinerve* polysaccharide can inhibit TGEV replication and alleviate ROS expression and apoptosis [58]. Tyrphostin A9 is a receptor tyrosine kinase inhibitor of the tyrphostin class. It can exert anti-TGEV effects by alleviating p38 activation and acts mainly in the post-adsorption stage [27]. The rhodamine derivative LJ001 is effective in inhibiting the proliferation of many enveloped viruses and can interpose the replication stage of TGEV life cycle [59]. Melatonin and structural analogues, including indole, tryptamine, and L-tryptophan, are adept at perturbing TGEV entry [60]. Surfactin from *Bacillus subtilis* can inactivate TGEV by acting directly on virions without disrupting viral integrity. It acts on viral lipids and increases the positive curvature of lipid monolayer, thereby inhibiting the fusion between viral and cellular membranes [61]. Tomatidine and 25HC also inhibit TGEV replication, but the mechanism of action remains to be elucidated. Meanwhile, they display antiviral activities against PEDV. Their inhibitory mechanisms on these two viruses may be similar, and their modes of action on PEDV will be discussed below [62,63].

### 3.2. Bioactive Compounds against PEDV

Tomatidine, a steroidal alkaloid extracted from the skin and leaves of tomatoes, can bind with PEDV 3CLpro and suppress 3CLpro activity, exhibiting excellent inhibitory effects on PEDV replication [62]. Quercetin is a flavonoid molecule that also targets PEDV 3CLpro [65], and quercetin 7-rhamnoside affects the initial stage of PEDV infection [66]. Glycyrrhizin, the main component of licorice root extracts, is a traditional medicine and HMGB1 inhibitor. It mainly intervenes in the entry and replication of PEDV and can restore the influence of PEDV on the HMGB1/TLR4-p38 pathway [26,67]. The addition of homoharringtonine at 0, 2 and 6 hpi significantly inhibits PEDV replication, and this inhibitory effect of homoharringtonine is related to its mitigation of eIF4E phosphorylation [68]. *Aloe* extract exerts an inhibitory effect at the late stage of the PEDV life cycle and can also directly inactivate virions without affecting the viral genome and S1 protein [69]. Epigallocatechin-3-gallate, the main polyphenol in green tea, functions in multiple stages of the PEDV life cycle, including attachment, entry, replication and assembly [70]. Polysaccharide from *Ginkgo biloba* exocarp can effectively alleviate PEDV-induced cytopathic effect by acting on viral attachment and entry [71]. Ergosterol peroxide restricts PEDV internalization, replication and release. In addition, it has the ability to inactivate PEDV virions and alleviate PEDV-induced apoptosis [11]. The aqueous leaf extract of the *Moringa oleifera* tree is able to suppress PEDV-induced oxidative stress (e.g., reduce ROS and malondialdehyde (MDA) production and restore glutathione peroxidase (GSH-Px) activity) and inhibit apoptosis. It has an inhibitory effect on the replication stage of PEDV life cycle [72]. Kwon et al. found that four phlorotannins isolated from *Ecklonia cava*, including eckol, 7-phloroeckol, phlorofucofuroeckol and dieckol, have good anti-PEDV activities. Among them, eckol, phlorofucofuroeckol and dieckol can potently inhibit hemagglutination and effectively interrupt viral attachment. Meanwhile, phlorofucofuroeckol and dieckol affect the viral replication step [73]. Cho et al. found that sabphenols A and B obtained from the leaves of *Sabia limoniacea* can inhibit PEDV replication and speculated that one of the mechanisms by which sabphenol B works is to inhibit PEDV 3CLpro activity [74]. *Pogostemon cablin* (Blanco) Benth. is often used as a traditional Chinese medicine to treat diarrhea, fever and more. Chen et al. extracted four polysaccharides from its dry overground parts and named them *Pogostemon cablin* polysaccharide (PCP) 1.1, PCP1.2, PCP2.1 and PCP2.2. All of them have anti-PEDV activities, especially PCP1.1. Among them, PCP1.1 and PCP1.2 mainly function in the replication stage of PEDV life cycle, while PCP2.1 and PCP2.2 affect PEDV penetration and replication [75]. Moreover, Kim et al. obtained five escin derivatives with low cytotoxicity and strong anti-PEDV activity from the seeds of *Aesculus turbinata* (Japanese horse chestnut) by two-step hydrolysis, including protoaescigenin, escinidin, aesculuside B and two new compounds (compounds 5 and 6) [76]. Chen et al. synthesized a series of phenanthridine derivatives and found that three of them (compounds 1, 2 and 4) had effective inhibitory activities against PEDV, which was associated with their inhibition of Hsc70 expression [49]. Another team designed 18 carbazole derivatives and identified two of them (No.7 and No.18) with low cytotoxicity and potent anti-PEDV effects [77]. Further studies showed that these two carbazole derivatives mainly inhibit viral attachment. Wang et al. identified that the ZINC natural product ZINC12899676 is able to inhibit PEDV NTPase activity by binding to its active site and changing its active pocket conformation [78]. Zhou et al. screened two compounds targeting PEDV 3CLpro, 3-(aminocarbonyl)-1-phenylpyridinium and 2,3-dichloronaphthoquinone, using a fluorescence resonance energy transfer-based assay [79]. A77 1726 is an active metabolite of leflunomide and can restrain PEDV replication by suppressing JAK and Src kinase activities [51]. Lithium chloride (LiCl) can thwart the entry and replication of PEDV and attenuate apoptosis [80]. Salinomycin is a monocarboxylic ionophore isolated from *Streptomyces albus*. It has the ability to restrict PEDV entry, interfere with the post-entry stage and alleviate the activation of MAPK pathways, including ERK1/2, JNK and p38 [82]. Since Yuan et al. found that surfactin has an inhibitory effect on PEDV, their team synthesized 10 surfactin analogues to explore drugs with high anti-PEDV activity and low hemolytic activity [61,83]. Among them, SLP5, a linear lipopeptide with three carboxyl groups, has a high selectivity index. It can act directly on PEDV. Caerin1.1 is a cationic amphibian antimicrobial peptide with 25-residues derived from the granular glands within the skin of the Australian green tree frog. It can destroy the integrity of the viral membrane and directly inactivate virions [84]. Griffithsin, a high-mannose-specific lectin from *Griffithsia* spp. marine red algae, has the property of intervening PEDV attachment and cell-to-cell transmission [85]. Xanthohumol, a small molecule from hops (*Humulus lupulus*), targets PEDV 3CLpro [87]. PEDV induces G0/G1-phase arrest to promote viral replication, while 1,25-dihydroxyvitamin D_3_ [1,25(OH)_2_D_3_] can regulate cell cycle progression through the ERK1/2 pathway, such as decreasing the G0/G1 phase and enlarging the S phase, thus exerting anti-PEDV effects. 1,25(OH)_2_D_3_ also ameliorates apoptosis and mitochondria damage caused by PEDV [88]. As mentioned above, cholesterol plays an important role in the attachment and internalization of PEDV. 25HC negatively regulates cholesterol production and can block PEDV entry [63]. Moreover, hypericin, proanthocyanidins from *Alpinia zerumbet*, *Epimedium koreanum* Nakai water extract, extracts of medicinal herbs Cimicifuga rhizoma, Meliae cortex, Coptidis rhizoma, Phellodendron cortex and Sophora subprostrata radix, tyrphostin A9, melatonin, indole, tryptamine, remdesivir (RDV), RDV nucleoside and β-_D_-N^4^-hydroxycytidine all have inhibitory effects on PEDV [27,56,60,89,90,91,92].

### 3.3. Bioactive Compounds against PDCoV

Ergosterol peroxide blocks PDCoV attachment and entry, and acts in the early and middle phases of the viral post-entry stage. Moreover, it can directly inactivate PDCoV infectivity and relieve apoptosis. Its antiviral activity is associated with its mitigation of PDCoV-induced p38 activation [25]. LiCl and diammonium glycyrrhizinate exert inhibitory effects at the early stage of PDCoV infection, and diammonium glycyrrhizinate can block viral attachment [81]. Both of them can alleviate PDCoV-induced apoptosis. RDV efficiently diminishes PDCoV replication in Huh7 cells but not in LLC-PK1 cells [93]. Scholars speculated that LLC-PK1 cells lack the cellular process required for RDV antiviral activity, which needs further research to elucidate. The anti-PDCoV effect of RDV in other cells and in vivo also needs to be validated. The inhibition of PDCoV by selenomethionine is related to its enhancement of cellular immunity (such as the upregulation of MAVS expression, IRF3 phosphorylation and IFN-α/β production) and antioxidant capacity (such as increased GSH-PX activity and SOD content and decreased H_2_O_2_ content) [94]. Griffithsin restricts PDCoV attachment and internalization by binding to S protein [86]. 25HC acts on the entry of PDCoV [64]. Considering that 25HC also has inhibitory effects on PEDV, TGEV and severe acute respiratory syndrome coronavirus 2 (SARS-CoV-2), we speculate that 25HC has the potential to be developed into a broad-spectrum anti-coronavirus drug [63,96]. Bile acids are produced from cholesterol oxidation in the liver and metabolized in the gut and are closely related to intestinal homeostasis. The bile acids chenodeoxycholic acid (CDCA) and lithocholic acid (LCA) have been shown to perturb the post-entry stage of PDCoV, which is related to their induction of IFN-λ3 and ISG15. Between them, LCA acts through the G protein-coupled receptor-IFN-λ3-ISG15 signaling axis [95]. However, some bile acids such as cholic acid and CDCA have been shown to promote the replication of PEDV and SADS-CoV [33,97]. Rhodanine derivative LJ001, melatonin, indole, tryptamine and L-tryptophan also restrict PDCoV infection [59,60]. LJ001 mainly targets the viral replication stage, and melatonin targets viral entry.

## 4. Conclusions

With the cross-species transmission and the emergence of new CoVs, CoVs have developed into a pathogen that seriously threaten human health. PECoVs are a long-term threat to agriculture. In addition, although reports of human infection with porcine viruses are uncommon, cases have appeared. A thorough understanding of the host cell factors required for the efficient replication of PECoVs will accelerate the refinement of CoV pathogenesis to cope with existing and even emerging CoVs. At present, there is no commercially available drug for the prevention and treatment of PECoV infection. Once the disease occurs, it is necessary to isolate the infected pigs in time, strengthen nursing, alleviate water loss, and prevent the secondary or complicating infection of other pathogenic microorganisms. The antiviral activity of some bioactive compounds described in this paper has been verified in vivo. Homoharringtonine, *Aloe* extract, *Epimedium koreanum* Nakai water extract, phenanthridine derivatives and surfactin can effectively protect piglets from PEDV infection, and ergosterol peroxide can protect piglets from PDCoV infection [49,61,68,69,90,98]. The systematic summary of agents showing antiviral activities against PECoVs not only provides references for the treatment of PECoVs but also provides suggestions for the development of agents against other CoVs.

## Figures and Tables

**Table 1:** Bioactive compounds with anti-PECoV activity and their possible action mechanisms.

Compound	Type of Virus	Reported Antiviral Mechanism	Reference
Curcumin	TGEV	Interferes with the early stage of viral infection and inactivates virions directly	[55]
Hypericin	TGEV	Inhibits 3CLpro activity	[56]
	PEDV	Inhibits 3CLpro activity	[56]
(+)-Catechin	TGEV	Alleviates ROS generation	[57]
*Polygonum Cillinerve* polysaccharide	TGEV	Reduces ROS expression and alleviates apoptosis	[58]
Tyrphostin A9	TGEV	Inhibits p38 activation Interferes with viral post-adsorption stage	[27]
	PEDV	/	[27]
Rhodamine derivative LJ001	TGEV	Interferes with the replication stage of viral life cycle	[59]
	PDCoV	Interferes with the replication stage of viral life cycle	[59]
Melatonin	TGEV	Blocks viral entry	[60]
	PEDV	Blocks viral entry	[60]
	PDCoV	Blocks viral entry	[60]
Indole	TGEV	Blocks viral entry	[60]
	PEDV	Blocks viral entry	[60]
	PDCoV	/	[60]
Tryptamine	TGEV	Blocks viral entry	[60]
	PEDV	Blocks viral entry	[60]
	PDCoV	/	[60]
l-tryptophan	TGEV	Blocks viral entry	[60]
	PDCoV	/	[60]
Surfactin	TGEV	Acts on viral lipids to inhibit fusion between viral and cellular membrane	[61]
	PEDV	/	[61]
Tomatidine	TGEV	/	[62]
	PEDV	Inhibits 3CLpro activity	[62]
25-hydroxycholesterol	TGEV	/	[63]
	PEDV	Blocks viral entry	[63]
	PDCoV	Blocks viral entry	[64]
Quercetin	PEDV	Inhibits 3CLpro activity	[65]
Quercetin 7-rhamnoside	PEDV	Affects the initial stage of viral infection	[66]
Glycyrrhizin	PEDV	Interferes with viral entry and replication stages Regulates HMGB1/TLR4-p38 pathway	[26,67]
Homoharringtonine	PEDV	Alleviates eIF4E phosphorylation	[68]
*Aloe* extract	PEDV	Affects the late stage of viral life cycle and inactivates virions directly	[69]
Epigallocatechin-3 gallate	PEDV	Inhibits viral attachment, entry, replication and assembly	[70]
Polysaccharide from *Ginkgo biloba* exocarp	PEDV	Inhibits viral attachment and entry	[71]
Ergosterol peroxide	PEDV	Inactivates virions directly and inhibits viral internalization, replication and release Alleviates PEDV-induced apoptosis	[11]
	PDCoV	Inhibits viral attachment, entry, and the early and middle phases of post-entry stage Alleviates apoptosis and elevated p38 phosphorylation	[25]
The aqueous leaf extract of the *Moringa oleifera* tree	PEDV	Interferes with the replication stage of viral life cycle Suppresses oxidative stress and alleviates apoptosis	[72]
Phlorotannins	PEDV	Phlorofucofuroeckol and dieckol block viral attachment by inhibiting hemagglutination, and interfere with the viral replication step Eckol blocks viral attachment by inhibiting hemagglutination	[73]
Sabphenols A and B	PEDV	Sabphenol B interacts with 3CLpro	[74]
*Pogostemon cablin* polysaccharide (PCP)	PEDV	PCP1.1 and PCP1.2 interfere with the replication stage of viral life cycle PCP2.1 and PCP2.2 interfere with viral penetration and replication stages	[75]
Escin derivatives	PEDV	/	[76]
Phenanthridine derivatives	PEDV	Inhibit Hsc70 expression	[49]
Carbazole derivatives	PEDV	Inhibit viral attachment	[77]
ZINC12899676	PEDV	Inhibits NTPase activity	[78]
3-(aminocarbonyl)-1-phenylpyridinium	PEDV	Inhibits 3CLpro activity	[79]
2,3-dichloronaphthoquinone	PEDV	Inhibits 3CLpro activity	[79]
A77 1726	PEDV	Inhibits JAK and Src kinase activities	[51]
LiCl	PEDV	Interferes with viral entry and replication stages Alleviates apoptosis	[80]
	PDCoV	Interferes with the early stage of viral infection Alleviates apoptosis	[81]
Salinomycin	PEDV	Interferes with viral entry and post-entry stagesAlleviates the activation of ERK1/2, JNK and p38	[82]
Surfactin analogue SLP5	PEDV	Acts directly on the virus	[83]
Caerin1.1	PEDV	Destroys the integrity of the viral membrane	[84]
Griffithsin	PEDV	Inhibits viral attachment and disrupts cell-to-cell transmission of virions	[85]
	PDCoV	Inhibits viral attachment and internalization by binding to S protein	[86]
Xanthohumol	PEDV	Inhibits 3CLpro activity	[87]
1,25-dihydroxyvitamin D_3_	PEDV	Regulates cell cycle progression through the ERK1/2 pathway Alleviates cell apoptosis and mitochondria damage	[88]
Proanthocyanidins from *Alpinia zerumbet*	PEDV	/	[89]
*Epimedium koreanum* Nakai water extract	PEDV	/	[90]
Extracts of medicinal herbs *Cimicifuga rhizoma*, *Meliae cortex*, *Coptidis rhizoma*, *Phellodendron cortex* and *Sophora subprostrata* radix	PEDV	/	[91]
Remdesivir (RDV)	PEDV	/	[92]
	PDCoV	/	[93]
Nucleoside analogs RDV nucleoside and β-d-N^4^-hydroxycytidine	PEDV	/	[92]
Diammonium glycyrrhizinate	PDCoV	Inhibits viral attachment Alleviates apoptosis	[81]
Selenomethionine	PDCoV	Enhances cellular immunity Improves antioxidant capacity	[94]
Chenodeoxycholic acid	PDCoV	Interferes with viral post-entry stage Promotes IFN-λ3 and ISG15 production	[95]
Lithocholic acid	PDCoV	Interferes with viral post-entry stage Promotes IFN-λ3 and ISG15 production through the G protein-coupled receptor-IFN-λ3-ISG15 axis	[95]

## Data Availability

Not applicable.
